# Diagnostic and treatment modalities for patients with cervical lymph node metastases of unknown primary site – current status and challenges

**DOI:** 10.1186/s13014-017-0817-9

**Published:** 2017-05-10

**Authors:** Jens Müller von der Grün, Aykut Tahtali, Shahram Ghanaati, Claus Rödel, Panagiotis Balermpas

**Affiliations:** 1 Department of Radiation Oncology, University Hospital, Johann Wolfgang Goethe University, Theodor Stern Kai 7, 60590 Frankfurt, Germany; 2 Department of Otolaryngology and Head and Neck Surgery, Johann Wolfgang Goethe University, Frankfurt, Germany; 3 Department of Maxillofacial Surgery, Johann Wolfgang Goethe University, Frankfurt, Germany; 4 German Cancer Research Center (DKFZ), Heidelberg, Germany; 5 German Cancer Consortium (DKTK), Frankfurt, Germany

**Keywords:** CUP, Cancer of unknown primary, Cervical, Lymph node, Head and neck cancer

## Abstract

**Background and Purpose:**

This review aims to provide a comprehensive overview of the literature and elucidate open questions for future clinical trials concerning diagnostics and treatment modalities for cervical cancer of unknown primary (CUP).

**Methods:**

A literature search for head and neck CUP was performed with focus on diagnostics and therapies as well as molecular markers.

**Results:**

High level evidence on CUP is limited. However, it seems that a consensus exists regarding the optimal diagnostic procedures. The correct implementation of biomarkers for patient stratification and treatment remains unclear. An even greater dispute dominates about the ideal treatment with publications ranging from sole surgery to surgery with postoperative bilateral radiotherapy with inclusion of the mucosa and concomitant chemotherapy.

**Conclusions:**

Cervical CUP represents a very heterogeneous malignant disease. On this account many aspects concerning treatment optimization remain unclear, despite a considerable number of publications in the past. Future research in form of prospective randomized trials is needed in order to better define patient stratification criteria and enable tailored treatment.

## Background

Cancer of unknown primary site (CUP) includes a various group of metastatic diseases whose primary tumor is not detected after clinical examination and extended diagnostic procedures. Reasons therefore may be involution or a slower growth rate at the primary tumor site, due to different genetic alterations in the primary or the metastases [1]. Dependent on the country, CUP represents 2–8% of the overall malignancies [2] and 3–5% of all solid tumors [3–5]. The estimated occurrence of CUP in the head and neck (HNCUP) region varies between 3 and 9%, with histological findings of a squamous cell malignancy in 53–77% of the cases [6–8]. The frequency of a subsequent mucosal emergence of the primary site in the head and neck region varies between 4 and 21% percent in the studies reviewed [9–28]. The most frequently encountered primary symptom is a cervical mass due to enlarged lymph nodes (94%) [15], mostly located in level 2 (30–50%), followed by level 1 and 3 (10–20%) and 4 and 5 (5–10%) [2, 15]. Bilateral involvement of the neck is reported in less than 10% of the cases [6, 8, 15, 18, 19, 29, 30]. When node metastases are found in levels 1-3, the primary site is suspected to be in the head and neck region. Upon affliction of the levels 4–5, the primary tumor most likely is located below the clavicles [31–33]. The time interval between noting the cervical mass and final diagnosis of HNCUP ranges from 2 to 5 months [6, 8, 34].

HNCUP patients are predominantly men, aged 55–65 years, showing typical risk factors for head and neck cancer such as tobacco and alcohol abuse [6, 8, 15, 18, 29, 35]. Patients with human papillomavirus (HPV, ~90% HPV-16), detected in lymph-node metastases represent a different and growing population [36] with a median age of at least five years less than HPV-negative patients, less tobacco and alcohol abuse and significant better prognosis [37, 38].

Since no prospective randomised studies are available for HNCUP patients, the therapeutic strategies for HNCUP differ widely and are based on retrospective studies, clinical experience and institutional policy. They range from surgery or (chemo)-radiotherapy alone to surgery plus adjuvant radiotherapy of various extents with or without concomitant chemotherapy [11, 29, 39, 40]. The prognosis for patients with CUP highly depends on the histology and involved region ranges from poor (adenocarcinoma metastatic to bone, brain and/or viscera) to favorable (e.g., squamous cell carcinoma metastatic to neck lymph nodes). The median survival of the poor prognostic group ranges from 7 to 11 months, whereas the survival of the favorable subset is similar to head and neck carcinomas with known primaries (e.g., HNSCC) [2, 41–43]. Here, we provide a comprehensive review of current diagnostic and therapeutic strategies, discuss open questions and challenges in the management of HNCUP patients like (stage dependent) uni- versus multimodality treatment, RT treatment volumes and the need of concomitant chemotherapy and also propose a treatment algorithm.

### Diagnostics: what should be considered standard and which are the implications of new molecular markers?

Clinical examination and diagnostic procedures aim at staging the tumor according to the UICC-TNM-classification system. HNCUP is a diagnosis of exclusion; not until after all workup is completed, the classification can be reduced to solely N and M defining CUP.

#### Patient history and examination

**Fig. 1:**
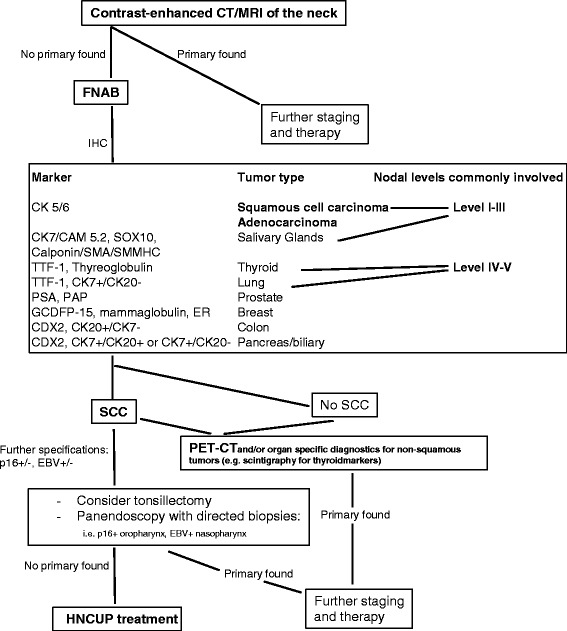
HNCUP diagnostics

If the patient history reflects excessive use of alcohol and tobacco, the primary site is unlikely to be situated in the nasopharynx, whereas promiscuity and orogenital contact suggest findings within the oropharynx. Also a history of skin lesions of the head and neck can guide the search [44]. The patient usually presents with a painless, unilateral cervical mass. Affliction of the levels 1–3 indicates the primary site to be located in the head and neck region, whereas a mass in levels 4–5 suggests the primary tumor situated at the lower neck (e.g., thyroid gland) or below the clavicles [31–33] (Fig. 1). Further examination is performed through exploring the head and upper aerodigestive tract using a nasopharyngoscope.

#### FNAB

FNAB (Fine-needle aspiration biopsy) of the cervical mass is the first and most commonly used diagnostic procedure, as it is minimal invasive and associated with a negligible risk of spreading the tumor along the needle’s path. After routine staining, the diagnostic sensitivity for metastatic neck lymph nodes ranges from 83 to 97% with a specifity of 91–100% when performed by an experienced histopathologist [45].

#### Immunohistochemistry

Immunohistochemistry (IHC) is an important tool to identify the tissue’s origin. General staining identifies cell morphologies and abnormal/malignant cell populations. Afterwards, an initial IHC-panel for broad cancer types including epithelial, melanocytic and lymphoid markers is used. General markers for carcinomas are the cytokeratins, i.e., intermediate filaments specific to epithelium. Markers for lymphomas are CLA – common leukocyte antigen, ALK1 – anaplastic lymphoma kinase, CD30 and CD43. For melanomas there are S-100, HMB45 – anti-human melanosome, and Melan-A [46]. In case of carcinoma, its subtype is evaluated by considering morphological aspects followed by specific antibodies, such as CK5, CK6, CK7 or TTF-1 [46]. The most common tumor types for HNCUP are squamous cell carcinomas SCC and adenocarcinomas.

#### Imaging

Available imaging techniques for patients with HNCUP are CT- and MRI- as well as 18F-fluoro2-deoxyglucose positron emission tomography (FDG-PET)-scans (Fig. 1). A quick, inexpensive, procedure with high spatial resolution is the contrast-enhanced CT-scan from the skull base to clavicles, complemented or substituted by a gadolinium contrast-enhanced MRI with superior soft tissue resolution [43]. In case of a cervical lymph node metastasis, the chance for CT, MRI or both to detect the primary site ranges from 9 to 23% [7, 47–49]. When suspicious findings on imaging are used to guide biopsy, the chance to find the primary tumor rises up to 60% [50]. For lymph nodes located in levels 4–5, additional chest/abdominal/pelvic CT-scans are recommended [51]. FDG-PET is a useful diagnostic tool when standard radiological work-up is completed with negative or inconclusive results and should be performed before any invasive procedures, which possibly hamper the evaluation of the scans due to iatrogenic induced tissue alteration [52–56]. Its capability for tumor detection is down to a size of ≥5 mm. Several studies and reviews adressed the additional benefits of FDG-PET for patients with HNCUP (Table 1) [54–56]. The extent of pre-FDG-PET diagnostic workup differs between the studies, so that it becomes difficult to compare the reported sensitivities and specifities and to quantify the additional value of PET [57].

**Table 1:** Reviews on FDG-PET techniques used for patients with HNCUP; NR – not reported; [54–56]

Review studies published (Year)	No. of Studies/Patients	Technique	Primary Tumor Detection Rate (%)	Sensitivity (%)	Specifity (%)	Highest false positive rate
Rusthoven et al., 2004 [54] (1994–2003)	16/302	FDG-PET	24.5	88.3	74.9	Tonsils (39.3%)
Kwee et al., 2009 [56] (2005–2007)	11/433	FGD-PET/CT	37	84.0	84.0	Oropharynx (15%) Lung (15%)
Al-Ibraheem et al., 2009 [55] (2000–2009)	8/180	FGD-PET FDG-PET/CT	28.3	NR	NR	NR overall 16.7%

#### Panendoscopy with biopsies

Panendoscopy of the upper aerodigestive tract (P-UADT), including naso-, oro- and hypopharynx as well as laryngoscopy and esophagoscopy, is performed under general anesthesia. Biopsies are taken from radiologically or clinically suspicious sites [43]. Additional bronchoscopy is recommended when indicated by imaging [44]. Repetition of panendoscopy is only indicated when directed biospy failed during the first procedure [50, 58]. Ipsilateral tonsillectomy leads to primary tumor detection in 18–44.6% of the cases. Waltonen et al. [47] reported the highest success rate for detection of the primary tumor by PET-CT scans plus panendoscopy with directed biopsies, with or without tonsillectomy (59.6%).

#### Molecular studies

HPV DNA, when found in metastasis, directs the search for the primary tumor to the oropharynx, as the prevalence of HPV in non-oropharyngeal squamous cell cancers currently is only 22%. HPV status can be determined by in-situ hybridization (ISH) or polymerase chain reaction (PCR), dectecting HPV DNA or by HPV E6/E7 RNA expression detected by quantitative reverse transcriptase-PCR (qRT-PCR). As a HPV surrogate marker, immunohistochemical staining of p16, a human tumor-suppressor protein [59–65], is also widely used. Despite showing a significantly improved disease-free survival, some authors like Dixon et al. could not find an improved overall survival for p16-positive HNCUP patients in their studies [66]. Other reports showed a significant positive impact of HPV/p16 only when combined with other factors like (non-)smoking [67]. A meta-analysis published in 2007 regarding non-oropharyngeal HNSCC shows congruent results [68]. However, most of the published literature agrees that HPV/p16 is a positive prognostic indicator for HNCUP [69, 70].

TP53 (protein name: p53) is a tumor-suppressor gene which is altered in about 50% of human malignancies, either by mutation or inactivation due to viral or cellular protein interactions leading to p53 degradation [71]. Significantly impaired outcome for patients with mutated p53 status or overexpression of p53 (whose expression directly correlates with the mutated protein, as the second tends to accumulate) in HNCUP and HNSCC has been demonstrated before [72, 73]. Some of the authors also examined the impact of the combined HPV/p53 status on survival and came to the conclusion that p53 could be an independent prognostic factor regardless of the HPV status [74].

Epstein-Barr virus (EBV) is consistently associated with nasopharyngeal carcinoma (NPC), especially with poorly or undifferentiated and nonkeratinizing types [75]. NPC is much more common in southern China and southeast Asia than in Europe or north America [74]. EBVs latent membrane protein 1 is highly suspicious of having a central role in both initiation and progression of the tumor [76–78]. EBV-DNA is routinely detected by PCR with sensitivity and specifity close to 90% from FNAB samples [79–82].

The data above suggest that the importance of detecting the HPV and EBV-DNA (or their surrogate proteins) in a metastatic lymph node in CUP-disease is high, as it can guide both further diagnostics and treatment (e.g., new directed biopsies or a radiotherapy-boost directed to the assumed primary tumor site) and also predict the patient’s outcome. These assays should be implemented in clinical routine for every HNCUP case. Immunohistochemistry for p53 is a simple and inexpensive method for further prognostic stratification and could be used as an additional prognostic parameter.

### Therapeutic options

Due to the lack of randomized trials, the optimal treatment strategies for HNCUP remain controversial. Therapeutic options depend on patient’s age, performace status, local extention, the site of the lymph node metastases and their histology. While tumor types other than SCC are often treated likewise cervical metastases with a known primary [83], this review focuses on the treatment of HNSCC-CUP. In former series, the HNCUP treatment aimed for the metastases as well as the suspected primary mucosal site. However, contemporary approaches need to evaluate the benefit of local neck and mucosal control separately, depending on the patient’s age and performance status. In early-stage neck disease, monomodal therapy is possible, whereas an advanced-stage neck disease usually requires an aggressive multimodal approach, comparable to locally advanced head and neck cancer [83]. Table 2 summarizes larger studies on HNCUP-therapy, including nodal stages of the patients treated, treatment modalities, radiotherapy and surgery specifications and finally control rates and survival data [9–28].

**Table 2:** Radiotherapy and outcome in major CUP studies

Study (Data Aquired) — No. of Patients (Total No. In Study)	Radiotherapy (No.) RT dosage median (range)	Nodal State No.(%)	Invasive Diagnostics — Surgical Treatment before RT No.(%)	locoregional control — distant metastases (median time to appearance, months)	overall survival — Head and Neck Mucosal Emergence No.(%) [most common site]
Bataini et al., 1987 [9] (1960–1980) — 138	dRT (90 pts): 70-80Gy pRT (48 pts): 50-60Gy bilateral 138, mucosa 43%	N1 45 (33) N2 32 (23) N3 60 (44) 137/138 EC 60%, UC 40%	FNAB/IB/EB 90 (65) — Adenectomy or RND 48 (35)	neck failure: definitive RT: 43% RND + RT: 17% ultimately — ultimately 25%	definitive RT: 22% RND + RT: 55% overall 33% at 5 years — 6 (4) [NR]
Jesse et al., 1973 [10] (1948–1968) — RT alone: 52 (210)	dRT (52): 50-60Gy + 5-10Gy unilateral 0, bilateral + mucosa 52	N1 12 (23) N2-3 23 (77) SCC 62%, UC 28%, GCSO 10%	EB 114/210 (52% total) — none in this group	initial local control 79% — NR	48% at 3 years — 3 (6) [oral cavity]
Weir et al., 1995 [11] (1970–1986) — 144	involved neck (85pts), bilateral + mucosa (59pts): 25 pts: 35-49Gy, 86 pts: 50-59Gy, 33 pts: 60-70Gy	N1 11 (5) N2 67 (47) N3 55 (38) Nx 5 (3) 138/144 UC 30%, SCC 62%, others 8%	EB 71 (50), IB 62 (43) FNAB 7 (7) — none	initial local control 51% — ultimately 23%	involved neck 37%, bilateral + mucosa 48%, overall 41%, at 5 years — 7 (5) [oropharynx]
Reddy et al., 1997 [12] (1974–1989) — 52	dRT (21): involved neck 70Gy (66–76) pRT (31): involved neck 64Gy (60–66) dRT/pRT: mucosa 60-66Gy, contralateral neck 46-50Gy unilateral 16, bilateral + mucosa 36	N1 9 (17) N2a 16 (31) N2b 7 (13) N2c 5 (10) N3 15 (29)	EB/IB 21 (40) — RND 31 (60)	NR — ultimately 15%	40% at 5 years — 10 (19) [base of tongue]
Colletier et al., 1998 [13] (1968–1992) — 136	pRT (136): involved neck 63Gy (34-70) mucosa + uninvolved neck 50-54Gy unilateral 16, bilateral 120	N1 31 (23) N2a 49 (37) N2b 25 (18) N2c 3 (2) N3 18 (13) Nx 10 (7) SCC 93%, NS 7%	EB 39 (29) — MND 64 (47) RND 33 (24)	84% with ECE 100% without ECE neck control at 5 years — 15% at 5 years	60% at 5 years 41% at 10 years — 14 (10) [oral cavity]
Fernández et al., 1998 [14] (1976-1996) — 67	dRT (3)/pRT (64): 50Gy bilateral + mucosa 67	N1 9 (13) N2 33 (49) N3 25 (37)	FNAB 40 (60) — RND 50 (75) MND 14 (21)	34% neck recurrence, mean 5 months — 22%, mean 17 months	22% at 5 years 20% at 10 years — 10 (15) [oropharynx]
Grau et al., 2000 [15] (1975-1995) — RT ± ND 250 (352)	dRT (250): neck 59Gy (28-93) neck + mucosa 66Gy (20-79) unilateral 26, bilateral + mucosa 224	N1 37 (15) N2 119 (48) N3 93 (37) Nx 1 (.4)	FNAB (12) CB (1) EB/IB (85) — ND (2)	local control 44% neck control 51% muscosal control 81% — NR	36% — 17 (7) [oropharynx]
Iganej et al., 2002 [16] (1969-1994) — 106	dRT: 66Gy (48-70) pRT: 60Gy (50-70) unilateral 16, bilateral + mucosa 163	N1 14 (13) N2a 27 (25) N2b 39 (37) N2c 2 (2) N3 24 (23)	EB alone 12 EB + RT 15 — RND 29 RT alone 24 RND + RT 26	54% neck recurrence, median 7 months; ultimately 34% neck failure — 9% (4)	53% at five years — 19 (18) [tonsil]
Yalin et al., 2002 [17] (1976-1988) — 114	UC: RT 50-70Gy SCC: RT 40-60Gy AC: 60Gy	N1 33 (29) N2 51 (45) N3 30 (26) UC 62%, SCC 24%, AC 14%	FNAB 111/EB 3 (100) — RND in case of bilateral disease (+thyrectomy in case of AC)	NR — NR	UC: 32%, SCC: 33% AC: 38% at 5 years — 12 (11) [tonsillar fossa]
Aslani et al., 2007 [18] (1987-2002) — 61	dRT (40): 64Gy (60-70) pRT (21): 60Gy (56-66) unilateral 11, bilateral 50	N1 16 (26) N2a 18 (31) N2b 13 (21) N2c 7 (11) N3 7 (11) UC 15%	FNAB 9 (15) EB 32 (52) — MND 9 (15) RND 11 (18)	neck control: with biopsy 76%, ND 85% at 5 years; 73% at 8 years — 11.5% (2-24 months)	79% at 5 years 67% at 8 years — 4 (7) [base of tongue]
Boscolo-Rizzo et al., 2006 [19] (1980-2001) — 82	dRT (32 pts), pRT (47 pts): 60-70Gy before 1989; 50Gy + 10-20Gy after 1989 unilateral 37, bilateral 42	N1 10 (12) N2a 14 (17) N2b 23 (28) N2c 3 (4) N3 32 (39)	FNAB/EB 82 (100) — RND 46 (56) MND 4 (5)	NR — 23% at 10 years	25% at 5 years 19% at 10 years — 10 (12) [base of tongue]
Beldi et al., 2007 [20] (1980-2004) — 113 (22/113 palliative intent)	dRT (59), pRT (54): involved neck 50Gy*, 60Gy** bilateral neck + mucosa 50Gy*, 56Gy** unilateral 45, bilateral 67	N1 21 (19) N2a 12 (11) N2b 42 (37) N2c 10 (9) N3 28 (25) SCC 77%, AC 5%, UC 10%, others 8%	FNAB 14 (12) EB 37 (33) — ND 62 (55)	disease free survival 27% at 5 years — NR	41% at 5 years — 19 (17) [NR]
Patel et al., 2007 [21] (1987-2006) — 70	pRT (60 pts): 50Gy or 54-60Gy unilateral 49, bilateral 11	N1 5 (7) N2a 13 (19) N2b 30 (43) N2c 4 (6) N3 18 (26)	FNAB 68 (97) EB 2 (3) — MND 70 (100)	ipsilateral control 84% contralateral control 93% at 5 years — 10% (9)	56% at 5 years — 8 (11) [base of tongue]
Corry et al., 2008 [22] (1998-2002) —	macroscopic disease 70Gy larger nodal masses 60Gy sites of suspected subclinical disease 50Gy	N2a 12 (12) N2b 43 (42) N2c 27 (26) N3 20 (20)	occult HNSCC\— ND 16 (16)	neck failure ultimately 9% — ultimately 23%	60% at 3 years (of 122 pts) —
Ligey et al., 2009 [29] (1990-2007) — 102 (122)	pRT (95): involved neck 66Gy mucosa + uninvolved neck 50Gy unilateral 59, bilateral 36	N1 9 (9) N2a 22 (23) N2b 33 (35) N3 15 (16) Nx 16 (17) EC 100%	EB 16 (17) — RND/MND 79 (83)	neck control 66% at 5 years — ultimately 30%	24% at 5 years — 9 (9) [tongue]
Lu et al., 2009 [23] (1989-2003) — 60	dRT (60): 69Gy (66-72) to mucosa; 62Gy (53-74) to involved areas; 51Gy (44-59) to uninvolved areas unilateral 25, bilateral 24, bilateral + mucosa 11	N1 10 (17) N2 39 (65) N3 11 (18)	FNAB 51 (85) EB 9 (15) — none	neck control 66% at 5 years — ultimately 18%	69% at 5 years — 10 (21) [nasopharynx]
Chen et al., 2011 [24] (2001-2009) — 60	dRT (15): involved neck 60-74Gy uninvolved neck 54-66Gy pRT (45): involved neck 63Gy (60-66) uninvolved neck 56Gy (54-60) bilateral + mucosa 60	N1 5 (8) N2a 26 (43) N2b 20 (33) N2c 0 (0) N3 9 (15)	FNAB 15 (25) EB 5 (8) — ND 8 (13) MND 32 (53)	locoregional control 89% at 2 years — 13% (9)	89% at 2 years — NR
Wallace et al., 2011 [25] (1964-2006) — 179	dRT (179): mucosa 57Gy (24-74) neck 65Gy (50-86) unilateral 5, bilateral + mucosa 174	N1 18 (10) N2a 48 (27) N2b 46 (26) N2c 11 (6) N3 56 (31)	none — ND 65 (36) before RT ND 44 (25) after RT	mucosal control 92% and neck control 81% at 5 years — 14% at 5 years	52% at 5 years — NR
Fakhrian et al., 2012 [26] (1988-2009) — 65	unilateral RT (17 pts): 60Gy (50-66) bilateral RT + mucosa (48 pts): 65Gy (28-70)	N1 14 (21) N2a 9 (14) N2b 34 (52) N2c 2 (3) N3 5 (8) UC 14%	EB 10 (15) — RND 27 (42) MND 24 (37)	locoregional lymph node recurrence, ultimately 14% — 28% (23)	48% at 5 years — 10 (15) [nasopharynx]
Tribius et al., 2012 [67] (2002-2011) — 63	dRT (63): involved neck 60-68Gy mucosa 60 Gy uninvolved neck 50-54Gy unilateral 7, bilateral 47	N1 6 (10) N2 38 (57) N3 19 (30)	FNAB 63 (100) — none	neck recurrence 25%, median 7 months — 13% (10)	76% at 2 years — 4 (6) [base of tongue]
Demiroz et al., 2014 [27] (1994-2009) — 41	dRT (19 pts): involved neck 70Gy univovled neck 50-59Gy pRT (22 pts): formerly inv. neck 60Gy (ECE 66Gy) univolved neck 54Gy bilateral + mucosa 67	N1 4 (10) N2a 10 (24) N2b 18 (44) N2c 0 (0) N3 9 (22)	FNAB/EB 41 (100) — RND 5 (12) MND 17 (41)	LRFS:dRT: 75% ND + RT: 76% at 4 years — definitve RT: 11% ND + RT: 28% ultimately	definitve RT: 85% ND + RT: 85% at 4 years — 2 (5) tonsillar fossa]
Straetmans et al., 2015 [28] (1997-2010) — 51	pRT (46 pts): univolved neck 50.4Gy or 46-50Gy involved neck 59.4-63Gy or 60-66Gy unilateral 32, bilateral 14	N1 4 (9) N2a 7 (15) N2b 28 (52) N3 12 (24)	FNAB (22) — MND 51 (100)	neck recurrence ultimately 18% — ultimately 18% (<24)	55% at 5 years — 0

Abbrevations: *UC* undifferentiated carcinoma, *AC* adenocarcinoma, *EC* epidermoid carcinoma, *GCSO* glandular carcinoma of salavary origin, *IB* incision biopsy, *CB* core biopsy, *EB* exicision biospy, *ND* neck dissection, *MND* modified neck dissection, *RND* radical neck dissection, *pts* patients, *dRT* definitive radiotherapy, *pRT* postoperative radiotherapy, *LRFS* locoregional relapse-free survival, *NS* not specified; *median dose in the 1980s, **median dose in the 1990s; TNM staging referring to UICC/AJCC classification actual when published; Tumor entity SCC if not described otherwise; adapted from references [9–28]

#### Is there a need for multimodality treatment for early-stage neck disease?

Early-stage HNCUP is defined as pN1 or mobile pN2a without extracapsular extention (ECE). Adequate regional control was reported both by studies performing surgery or radiotherapy (RT) as monotherapy. A bias exists, since usually patients with greater neck burden are treated more likely with RT primarily [10]. Although policy-dependend approaches prefer surgery alone with the option of salvage-RT [16, 84] or vice versa [15], there is some evidence for primary surgery: only pathology after surgery reliably proves ECE, which then necessitates RT with concomitant chemotherapy (CTx) and the vast majority of the patients in the published series implemented this approach (Table 2). In pN1 or pN2a situations without ECE, postoperative RT has not proven clear benefit regarding locoregional control or survival [85, 86]. However, some of the few studies addressing this topic are biased, due to their retrospective nature and the simplified statistics used [87, 88], so that the role of postoperative RT in these situations remains unclear. However, when RT was postponed and used for salvage treatment only, ultimate control above the clavicles still reached more than 90% in pN1 situations without ECE [84]. Surgery should also be followed by adjuvant RT in cases of connective tissue invasion (ECE), more than one involved node and a likelihood of residual microscopic disease in the neck (R1) [87, 88]. In cases without these risk factors postoperative RT could be considered.

#### How should advanced-stage neck disease be treated?

In advanced-stage neck disease (N2b-N3) a multimodal approach consisting of surgery and RT with or witout CTx is most common and seems to provide superior results regarding survival when compared to single-modality treatment [15, 18, 23]. This is true for the combination of surgery and RT compared to RT alone [25, 27, 89], as well as for surgery and RT compared to surgery alone, at least regarding the subsequent emergence of a primary tumor [15]. In cases of an unresectable bulk or unambiguously anticipated ECE/incomplete resection, primary chemoradiotherapy (CRT) is the treatment of choice in order to avoid excess toxicity from surgery and postoperative chemoradiotherapy. In most of the retrospective studies above, early-stage disease (1 small node involved) was surgically treated and unresectable masses primary irradiated, which could have biased the results and makes data interpretation difficult. However, due to the lack of prospective data, many crucial questions regarding the optimal radiotherapeutical treatment remain: uni- versus bilateral neck treatment, mucosal irradiation and the use of concomitant chemotherapy.

#### Which volumes should be irradiated?

In 2001, Nieder et al. [90] reviewed the management of HNCUP and reported results of various groups regarding ipsilateral versus mucosal and bilateral irradiation. Some results showed decreased tumor control and survival for ipsilateral therapy, while others failed to show any significant differences in outcomes between sole ipsilateral RT and comprehensive treatment of both neck sides and mucosa. When disease control was examined, there was no evidence supporting extended volume treatment over ipsilateral RT. The authors recommended a randomized trial between both options, but a similar trial was never accomplished: a prospective randomized trial (EORTC-24001-22005) starting in 2002 to compare ipsilateral versus bilateral plus mucosal irradiation in HNCUP failed to provide any results, due to very limited patient enrollment. Table 2 demonstrates that most of the larger studies included unilateral as well as bilateral treatment in varying proportions. However, no obvious outcome differences exist between those that treated predominantely unilateral (e.g., Straetmans et al., Patel et al.) and those who preffered a unilateral irradiation (e.g., Wallace et al., Fakhrian et al.), at least regarding overall survival. A recent large meta-analysis revealed no significant differences in 5-year-overall- and disease free survival (OS and DFS) between ipsi- and bilateral RT, but improved locoregional control and lower recurrence rates in favor of bilateral treatment [89]. When considering additional mucosal treatment (“presumed primary tumor”), recurrence rates were significantly lower and DFS better when extended radiation volumes where used, but no benefit for OS could be found and the improved locoregional control was associated with significantly higher severe toxicity [89].

In the current NCCN guidelines [91] no clear statement about the treatment volumes is being made, the approaches found in the literature vary and many of these data originate from the time before the routine use of PET and tonsillectomy. As diagnostic workup became more comprehensive, it could be shown that the numbers of patients developing primary site tumor is lower than indicated in previous literature [15] and also about twofold lower than the risk for nodal recurrence or distant metastases [90]. It seems that metastatic disease in general is nowadays the most common pattern of failure [92, 93], so that a possible benefit of a slightly improved locoregional control through extended volume radiotherapy can not be translated in an improved survival [84].

The advent of intensity modulated radiotherapy (IMRT) made the more sophisticated selection of the irradiation volumes essential, as it allows both sparing of organs at risk as well as missing the primary that would had been accidentally treated using older techniques. Previously, HNSCC/HNCUP was treated using a three-field technique including all mucosal sites and both sites of the neck [94], whereas today’s standard is intensity- IMRT preserving salivatory tissues [95]. The vast majority of the data presented here (Table 2) have been generated with older, non-conformal techniques. However, a possible strategy in modern series treating HNCUP could be the irradiation of selected mucosal sites, e.g., base of tongue for HPV-positive non-smokers or nasopharynx for EBV-positive non-smokers with nonkeratinizing subtypes and/or patients with Asiatic origin. Such approaches have become more common in the IMRT-era and the first data are encouraging [84, 96].

An overview of the radiation doses and treatment volumes in the greatest series published can be found in Table 2.

#### Is there a benefit for concomitant chemotherapy?

The value of adding chemotherapy to RT both in the definitive as in the postoperative setting for treating HNCUP patients remains unclear, despite its common use in many institutions [27, 29, 67, 97, 98]. Cisplatin (e.g., 100 mg/m^2^, days 1, 22 and 43) is the agent most frequently used in these cases [24, 99]. Established indications for concomitant chemotherapy in HNSCC are the definitive treatment of locally advanced tumors (e.g., a cT2cN2b tumor) or the postoperative treatment of high-risk tumors (e.g., a pT1pN3b tumor: extracapsular extension). Implementing chemotherapy for a HNCUP with one or more involved nodes after neck dissection would assume that it has a similar prognosis with such cases. This does not seem justified, since a cT1 tumor (in this case not detected, therefore CUP) generally has an excellent prognosis with RT alone [84, 100]. A recent study by Hosni et al. revealed an almost identical prognosis for patients with HNCUP and those with T1 base-of-tongue carcinoma [101]. These data would imply that both diseases may be treated the same way, i.e., without the use of chemotherapy. In a retrospective analysis examining the effect of concomitant cisplatin, involving 60 HNCUP-patients, no clear advantage could be found for the addition of chemotherapy and severe toxicities (grade 3+) occurred significantly more often [24]. Furthermore, in the era of HPV/p16 stratification a de-escalation of treatment and an alternative staging for positive tumors are already under discussion because of the distinct improved outcomes of this collective [102, 103]. The current paradigm for the indications for postoperative chemo-irradiation (R1, pN3b) originates from the pre-HPV-stratification era [104, 105]. Keller et al. [70] have conducted an analysis of clinicopathological data, including p16 and extracapsular extension (ECE), in HNCUP and could demonstrate a very similar prognosis in patients with or without ECE, even without chemotherapy, but the patient numbers in this analysis where very limited and so no safe conclusions can be drawn. A treatment-deescalation for HPV/p16 non-smokers could be imaginable, either through omitting chemotherapy or even by using chemotherapy in order to reduce RT-dose, following the paradigm of current HNSCC trials [102]. Table 3 shows the largest published studies implementing chemotherapy and the agents used in each case [10, 11, 53, 76, 77, 79–85].

**Table 3:** Concomitant Chemotherapy

Study	Concomitant chemotherapy No.(%)	Locoregional control	Overall survival
Yalin, 2002	UC, SCC: COP or PCV AC: PCV 114 (100)	NR	UC: 32%, SCC: 33% AC: 38% at 5 years
Boscolo-Rizzo, 2006	Platinum based 9 (11)	NR	25% at 5 years, 19% at 10 years
Beldi, 2007	Platinum based 21 (19)	disease free survival 27% at 5 years	41% at 5 years
Corry, 2008	Platinum based 102 (100)	neck failure ultimately 9%	60% at 3 years
Ligey, 2009	Platinum based 43 (45)	neck control 66% at 5 years	24% at 5 years
Lu, 2009	Platinum based 14 (23)	neck control 66% at 5 years	69% at 5 years
Chen, 2011	Platinum based 32 (53)	locoregional control 89% at 2 years	89% at 2 years
Wallace, 2011	Ctx (drugs NR) 13 (7)	mucosal control 92% and neck control 81% at 5 years	52% at 5 years
Fakhrian, 2012	Ctx 19 (29) Cis based 10, 5-FU + MMC 9	locoregional lymph node recurrence, ultimately 14%	48% at 5 years
Tribius, 2012	Cis 38 (60)	neck recurrence 25%, median 7 months	76% at 2 years
Demiroz, 2013	Ctx, 4 regimes 25 (61)	LRFS: dRT: 75% ND + RT: 76% at 4 years	definitve RT: 85% ND + RT: 85% at 4 years
Straetmans, 2014	Carbo 8 (16)	neck recurrence ultimately 18%	55% at 5 years

*Abbrevations*: *SCC* squamous cell carcinoma, *UC* undifferentiated carcinoma, *AC* adenocarcinoma, *EC* epidermoid carcinoma, *Ctx* chemotherapy, *Cis* cisplatin, *5FU* 5-flurouracil, *MMC* mitomycin C, *Carbo* carboplatin, *COP* cyclophosphamide, vincristine, prednisolone, *PCV* cisplatin, cyclophosphamide, vincristine [10, 11, 53, 76, 77, 79–85]

### Treatment algorithm

**Fig. 2:**
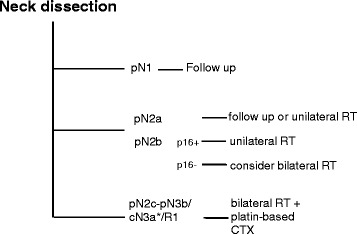
HNCUP therapy algorithm. RT: radiotherapy, CTX: chemotherapy, *inoperable

Based on the above considerations we tried to summarize the existing experience and develop a treatment proposal for further evaluation in a prospective mode (Fig. 2).

## Conclusions

To the best of our knowledge, no prospective phase III trial investigating treatment optimization for HNCUP currently exists. Treatment of cervical cancer of unknown primary remains a diagnostic and therapeutical challenge. Several improvements in instrument-based and pathological diagnostics have led to better understanding of this rare disease and less common missing of an undetected primary tumor. Multimodality treatment seems to provide superior results, especially for N2b-N3b cases. Until today, there is no unambiguous evidence of a survival benefit through treatment intensification with extended radiotherapy volumes and/or the implementation of concurrent chemotherapy. These questions can only be answered with the help of large prospective trials. Novel molecular parameters like the HPV-status will help stratifying patients for such trials and allow more valid results.

## Abbreviations

CRT: Chemoradiotherapy
CT: Computer tomography
CTx: Chemotherapy
CUP: Cancer of unknown primary
DFS: Disease free survival
EBV: Epstein-Barr virus
ECE: Extracapsular extention
FDG-PET: 18F-fluoro2-deoxyglucose positron emission tomography
FNAB: Fine-needle aspiration biopsy
HNCUP: CUP in the head and neck region
HNSCC: Head and neck squamous cell carcinoma
HPV: Human papilloma virus
IHC: Immunohistochemistry
IMRT: Intensity modulated radiotherapy
MRI: Magnet resonance imaging
NCCN: National Comprehensive Cancer Network
NPC: Nasopharyngeal carcinoma
OS: Overall survival
P-UADT: Panendoscopy of the upper aerodigestive tract
qRT-PCR: quantitative reverse transcriptase-PCR
RT: Radiotherapy
SCC: Squamous cell carcinoma
UICC: Union Internationale Contre le Cancer
